# Supplementary material for: Analysis of the Phenolic Profile of Chelidonium majus L. and Its Combination with Sericin: Balancing Antimicrobial Activity and Cytocompatibility
Source: Int J Mol Sci. 2025 Oct 11;26(20):9911. doi: 10.3390/ijms26209911 (PMC12563533; doi:10.3390/ijms26209911)
Supplement: Supplementary file 1 [file ijms-26-09911-s001.zip › ijms-3879091-supplementary.pdf]

Table S1. Standard curves of polyphenols.

Groups	Phenolic compound	Molecular formula	Retention time (min)	Slope	Interception
**Phenolic Acids**					
**Hydroxybenzoic acids**	4-H-phenylacetic acid	C8H8O3	7,52	297332	121827
**Hydroxycinnamic acids**	Dihydrocaffeic acid	C9H10O4	6,95	6009293	-10313439
	Caffeic acid	C9H8O4	7,57	19368608,03	-602411,1406
	Vanillic acid	C8H8O4	7,74	286731,6884	37941,43523
	Homovanillic acid	C9H10O4	8,28	1072846	137703
	Ferulic acid	C10H10O4	8,8	8769769,665	-39720,68738
	4,5-Dicaffeoylquinic acid	C25H24O12	9,01	6009293	-10313439
**Flavonoids**					
**Flavanol**	Catechin	C15H14O6	6,98	12822638,66	-208241,9256
	Epicatechin	C15H14O6	7,12	11616785,88	-2451397,702
**Flavanone**	Hesperidin	C28H34O15	9,01	106509,3728	1190,320508
	Naringenin	C15H12O5	11,32	106509,3728	1190,320508
	Hesperetin	C16H14O6	11,57	106509,3728	1190,320508
**Flavone**	Vitexin	C21H20O10	8,28	75438765,11	-854117,2976
	Cynaroside	C21H20O11	8,44	75438765	-854117
	Diosmin	C28H32O15	8,87	939692	26635
	Apigetrin	C21H20O10	9,01	75438765,11	-854117,2976
	Diosmetin-7-glucoside	C22H22O11	9,18	939692	26635
	Luteolin/Scutellarein	C15H10O6	10,38	75438765,11	-854117,2976
	Apigenin	C15H10O5	11,23	75438765,11	-854117,2976
	Diosmetin	C16H12O6	11,4	31792003	151871,406
	Rutin	C27H30O16	8,12	8806693	-2057338
	Kaempferol 3-rutinoside	C27H30O15	8,53	16379922	4841701

	Guaiaverin/Reynoutrin	C20H18O11	8,73	20404174	32246
	Quercitrin/Quercetin 3-rhamnoside	C21H20O11	8,84	27953311,22	-2782865,752
	Isorhamnetin-3-glucoside	C22H22O12	8,89	26585869	1103466
	Quercetin	C15H10O7	10,44	27953311,22	-2782865,752
	Kaempferol	C15H10O6	11,37	52119731,13	-2945639,274
	Isorhamnetin	C16H12O7	11,53	28940575,27	-12243096,79
**Others**					
**Phenolic Aldehyde**	Vanillin	C8H8O3	8,56	1123461	114469
**Phenylethanoids**	Hydrotyrosol	C8H8O3	8,56	1976426	133564
**Phenylpropanoids**	Phenylacetic acid	C8H8O2	9,88	15323	60984
